# Supplementary material for: Surrounded by Sound: The Impact of Tinnitus on Musicians
Source: Int J Environ Res Public Health. 2021 Aug 27;18(17):9036. doi: 10.3390/ijerph18179036 (PMC8431046; doi:10.3390/ijerph18179036)
Supplement: Supplementary file 1 [file ijerph-18-09036-s001.zip › ijerph-1321923-supplementary.pdf]

Music to your ears? The impact of tinnitus on professional musicians

Hello and welcome!

This is a questionnaire to explore the impact of tinnitus on professional musicians. It is being conducted by the British Tinnitus Association in collaboration with University of Nottingham and funded by Help Musicians. This study has been reviewed and given a favourable opinion by the University of Nottingham, Faculty of Medicine & Health Sciences Research Ethics Committee (ref: 471--2001).

This questionnaire will take approximately 15 minutes to complete and is made up of 4 sections: your eligibility, information about you, information about your tinnitus, and information about the impact of tinnitus on musicians' lives.

Thank you for your interest in this research and for offering to share your opinions and experiences about this important topic.

INSTRUCTIONS:
1. Please read the participant information sheet for full details on this project.
2. If you are happy to take part in this study, please complete the eligibility questions.
3. If you are eligible to take part, please continue to answer the general information questions (please note, these are not essential to completing the rest of the questionnaire).
4. Please select one answer for each question on the Tinnitus Functional Index to provide an overview of your tinnitus.
5. Finally, please provide detailed responses about your experience of living with tinnitus and its impact on you as a musician.

All information you provide in this questionnaire will be treated confidentially.

* 1. Have your read the participant information sheet?
Click here for participant information sheet.

- ○ Yes
- ○ No

* 2. I understand that...

- ☐ that my participation is voluntary and I can end the study at any time and withdraw my data by clicking the EXIT button.
- ☐ that my answers will be anonymous.
- ☐ the overall anonymized data from this study may be used in the future for research (with research ethics approval) and teaching purposes.

* 3. I agree to take part in this study

- ○ Yes
- ○ No

Music to your ears? The impact of tinnitus on professional musicians

Are you eligible to take part?

If you answer 'yes' to ALL of the following 4 questions, please complete the rest of this questionnaire.

If you answer 'no' to ANY of the following 4 questions, then you are not eligible to take part, but we thank you for your time.

The information you provide in this questionnaire will be treated confidentially.

* 4. Are you a professional musician? (Participants can be a solo artist, songwriter, composer, group member, artist/producer or a multidisciplinary artist, and of all genres).

◯ Yes

◯ No

* 5. Have you ever experienced tinnitus (ringing or buzzing in your ears/head)?

◯ Yes

◯ No

* 6. Do you reside primarily in the UK and are you eligible to work here?

◯ Yes

◯ No

* 7. Are you aged 18 or over?

◯ Yes

◯ No

Music to your ears? The impact of tinnitus on professional musicians

General information about you...

PURPOSE: You will be asked some questions to provide us with some general information about you.

INSTRUCTIONS: Please answer by selecting the response that most suits you. Please note that these questions are optional, you are not required to complete these questions in order to complete the rest of the questionnaire.

The information you provide in this questionnaire will be treated confidentially.

8. What is your date of birth? (dd/mm/yy)

Date

Date

DD/MM/YYYY

9. Which gender identity do you most identify with?

- Female
- Prefer not to say
- Male
- Prefer to self-describe:

10. What is your sexual orientation?

- Bi
- Heterosexual/straight
- Gay Man
- Prefer not to say
- Gay Woman/Lesbian
- Prefer to self-describe:

11. Choose one option that best describes your ethnic group or background.

12. What type of musician are you?

- solo artist
- group member
- songwriter
- artist/producer
- composer
- multidisciplinary artist
- Other (please specify)

13. How often do yo use hearing protection at work?

- Always
- Rarely
- Usually
- Never
- Sometimes

14. How long have you had tinnitus?

- 1 month or less
- 11-20 years
- 1 year or less
- 21-30 years
- 2-5 years
- 30+ years
- 6-10 years

Information about your tinnitus

Tinnitus Functional Index
PURPOSE: The following questions are used to measure the severity and impact of your tinnitus.

INSTRUCTIONS: Please answer the questions as accurately as possible as this is very Important to the outcome of this research. Please read each question below carefully. Please answer every question. To answer a question, select ONE of the answers that are listed for that question.
The information you provide in this questionnaire will be treated confidentially.

* 15. Over the PAST WEEK what percentage of your time awake were you consciously AWARE OF your tinnitus?

- 0% ◄ *Never aware*
- 10%
- 20%
- 30%
- 40%
- 50%
- 60%
- 70%
- 80%
- 90%
- 100% ◄ *Always aware*

* 16. Over the PAST WEEK how STRONG or LOUD was your tinnitus?

- 0 ◄ *Not at all strong or loud*
- 1
- 2
- 3
- 4
- 5
- 6
- 7
- 8
- 9
- 10 ◄ *Extremely strong or loud*

* 17. Over the PAST WEEK what percentage of your time awake were you ANNOYED by your tinnitus?

- 0% ◄*None of the time*
- 10%
- 20%
- 30%
- 40%
- 50%
- 60%
- 70%
- 80%
- 90%
- 100% ◄ *All of the time*

* 18. Over the PAST WEEK did you feel IN CONTROL in regard to your tinnitus?

- 0 ◄ *Very much in control*
- 1
- 2
- 3
- 4
- 5
- 6
- 7
- 8
- 9
- 10 ◄ *Never in control*

* 19. Over the PAST WEEK how easy was it for you to COPE with your tinnitus?

- 0 ◄ *Very easy to cope*
- 1
- 2
- 3
- 4
- 5
- 6
- 7
- 8
- 9
- 10 ◄ *Impossible to cope*

* 20. Over the PAST WEEK how easy was it for you to IGNORE your tinnitus?

- 0 ◄ *Very easy to ignore*
- 1
- 2
- 3
- 4
- 5
- 6
- 7
- 8
- 9
- 10 ◄ *Impossible to ignore*

* 21. Over the PAST WEEK how much did your tinnitus interfere with your ability to CONCENTRATE?

- 0 ◄ *Did not interfere*
- 1
- 2
- 3
- 4
- 5
- 6
- 7
- 8
- 9
- 10 ◄ *Completely interfered*

* 22. Over the PAST WEEK how much did your tinnitus interfere with your ability to THINK CLEARLY?

- 0 ◄ *Did not interfere*
- 1
- 2
- 3
- 4
- 5
- 6
- 7
- 8
- 9
- 10 ◄ *Completely interfered*

* 23. Over the PAST WEEK how much did your tinnitus interfere with your ability to FOCUS ATTENTION on other things besides your tinnitus?

- 0 ◄ *Did not interfere*
- 1
- 2
- 3
- 4
- 5
- 6
- 7
- 8
- 9
- 10 ◄ *Completely interfered*

* 24. Over the PAST WEEK how often did your tinnitus make it difficult to FALL ASLEEP or STAY ASLEEP?

- 0 ◄ *Never had difficulty*
- 1
- 2
- 3
- 4
- 5
- 6
- 7
- 8
- 9
- 10 ◄ *Always had difficulty*

* 25. Over the PAST WEEK how often did your tinnitus cause you difficulty in getting AS MUCH SLEEP as you needed?

- 0 ◄ *Never had difficulty*
- 1
- 2
- 3
- 4
- 5
- 6
- 7
- 8
- 9
- 10 ◄ *Always had difficulty*

* 26. Over the PAST WEEK how much of the time did your tinnitus keep you from SLEEPING as DEEPLY or as PEACEFULLY as you would have liked?

- 0 ◄ *None of the time*
- 1
- 2
- 3
- 4
- 5
- 6
- 7
- 8
- 9
- 10 ◄ *All of the time*

* 27. Over the PAST WEEK, how much has your tinnitus interfered with your ability to HEAR CLEARLY?

- 0 ◄ *Did not interfere*
- 1
- 2
- 3
- 4
- 5
- 6
- 7
- 8
- 9
- 10 ◄ *Completely interfered*

* 28. Over the PAST WEEK, how much has your tinnitus interfered with your ability to UNDERSTAND PEOPLE who are talking?

- 0 ◄ *Did not interfere*
- 1
- 2
- 3
- 4
- 5
- 6
- 7
- 8
- 9
- 10 ◄ *Completely interfered*

* 29. Over the PAST WEEK, how much has your tinnitus interfered with your ability to FOLLOW CONVERSATIONS in a group or at meetings?

- 0 ◄ *Did not interfere*
- 1
- 2
- 3
- 4
- 5
- 6
- 7
- 8
- 9
- 10 ◄ *Completely interfered*

* 30. Over the PAST WEEK, how much has your tinnitus interfered with your QUIET RESTING ACTIVITIES?

- 0 ◄ *Did not interfere*
- 1
- 2
- 3
- 4
- 5
- 6
- 7
- 8
- 9
- 10 ◄ *Completely interfered*

* 31. Over the PAST WEEK, how much has your tinnitus interfered with your ability to RELAX?

- 0 ◄ *Did not interfere*
- 1
- 2
- 3
- 4
- 5
- 6
- 7
- 8
- 9
- 10 ◄ *Completely interfered*

* 32. Over the PAST WEEK, how much has your tinnitus interfered with your ability to enjoy "PEACE AND QUIET"?

- 0 ◄ *Did not interfere*
- 1
- 2
- 3
- 4
- 5
- 6
- 7
- 8
- 9
- 10 ◄ *Completely interfered*

* 33. Over the PAST WEEK, how much has your tinnitus interfered with your enjoyment of SOCIAL ACTIVITIES?

- 0 ◄ *Did not interfere*
- 1
- 2
- 3
- 4
- 5
- 6
- 7
- 8
- 9
- 10 ◄ *Completely interfered*

* 34. Over the PAST WEEK, how much has your tinnitus interfered with your ENJOYMENT OF LIFE?

- 0 ◄ *Did not interfere*
- 1
- 2
- 3
- 4
- 5
- 6
- 7
- 8
- 9
- 10 ◄ *Completely interfered*

* 35. Over the PAST WEEK, how much has your tinnitus interfered with your RELATIONSHIPS with family, friends and other people?

- 0 ◄ *Did not interfere*
- 1
- 2
- 3
- 4
- 5
- 6
- 7
- 8
- 9
- 10 ◄ *Completely interfered*

* 36. Over the PAST WEEK, how often did your tinnitus cause you to have difficulty performing your WORK OR OTHER TASKS, such as home maintenance, school work, or caring for children or others?

- 0 ◄ *Never had difficulty*
- 1
- 2
- 3
- 4
- 5
- 6
- 7
- 8
- 9
- 10 ◄ *Always had difficulty*

* 37. Over the PAST WEEK, how ANXIOUS or WORRIED has your tinnitus made you feel?

- 0 ◄ *Not at all anxious or worried*
- 1
- 2
- 3
- 4
- 5
- 6
- 7
- 8
- 9
- 10 ◄ *Extremely anxious or worried*

* 38. Over the PAST WEEK, how BOTHERED or UPSET have you been because of your tinnitus?

- 0 ◄ *Not at all bothered or upset*
- 1
- 2
- 3
- 4
- 5
- 6
- 7
- 8
- 9
- 10 ◄ *Extremely bothered or upset*

* 39. How DEPRESSED were you because of your tinnitus?

- 0 ◄ *Not at all depressed*
- 1
- 2
- 3
- 4
- 5
- 6
- 7
- 8
- 9
- 10 ◄ *Extremely depressed*

Further information about your experience of tinnitus

Purpose: We want to understand in detail how tinnitus impacts on professional musicians.

Instructions: You will be asked about your tinnitus in more detail. Please answer each question with as much detail as possible. Please write 'NA' if the question is not applicable to you.

The information you provide in this questionnaire will be treated confidentially.

* 40. As well as tinnitus (also known as ringing in your ears), what conditions/illnesses (physical or mental health) have you been diagnosed with?

* 41. How do you think you got tinnitus?

* 42. Did you receive any information about tinnitus during your education?

- Yes
- No

If yes, can you tell me more about it?

If no, do you think it would have been helpful, and how?

* 43. Did you receive any information about tinnitus during any period of employment?

- Yes
- No

If yes, can you tell me more about it?

If no, do you think it would have been helpful, and how?

* 44. How does your tinnitus have an impact on your work life?

* 45. How does your tinnitus have an impact on your life outside of work?

* 46. Have you previously accessed healthcare/treatment or other forms of support for tinnitus? If so, could you please provide details?

* 47. What healthcare/treatment or other form of support do you think would be especially helpful for you and other musicians?

* 48. Do you have support for your tinnitus from those around you (e.g. family and/or friends)? If so, what do you find helpful?

49. Any other comments?

Thank you!

Thank you for taking part in this important research!

If you would like more information about the project, or would like to discuss taking part in the research, please call Georgie on 0114 250 9933 or email georgina@tinnitus.org.uk

If you would like general information about tinnitus please call the British Tinnitus Association helpline on 0800 018 0527.

British Tinnitus Association, Ground Floor, Unit 5, Acorn Business Park,
Woodseats Close,
Sheffield,
S8 0TB.
Registered charity number 1011145.
Office Tel: 0114 250 9933| Email: georgina@tinnitus.org.uk
Web: www.tinnitus.org.uk/musiciansquestionnaire
